# Supplementary figures and images for: The metastable brain associated with autistic-like traits of typically developing individuals
Source: PLoS Comput Biol. 2021 Apr 16;17(4):e1008929. doi: 10.1371/journal.pcbi.1008929 (PMC8081345; doi:10.1371/journal.pcbi.1008929)

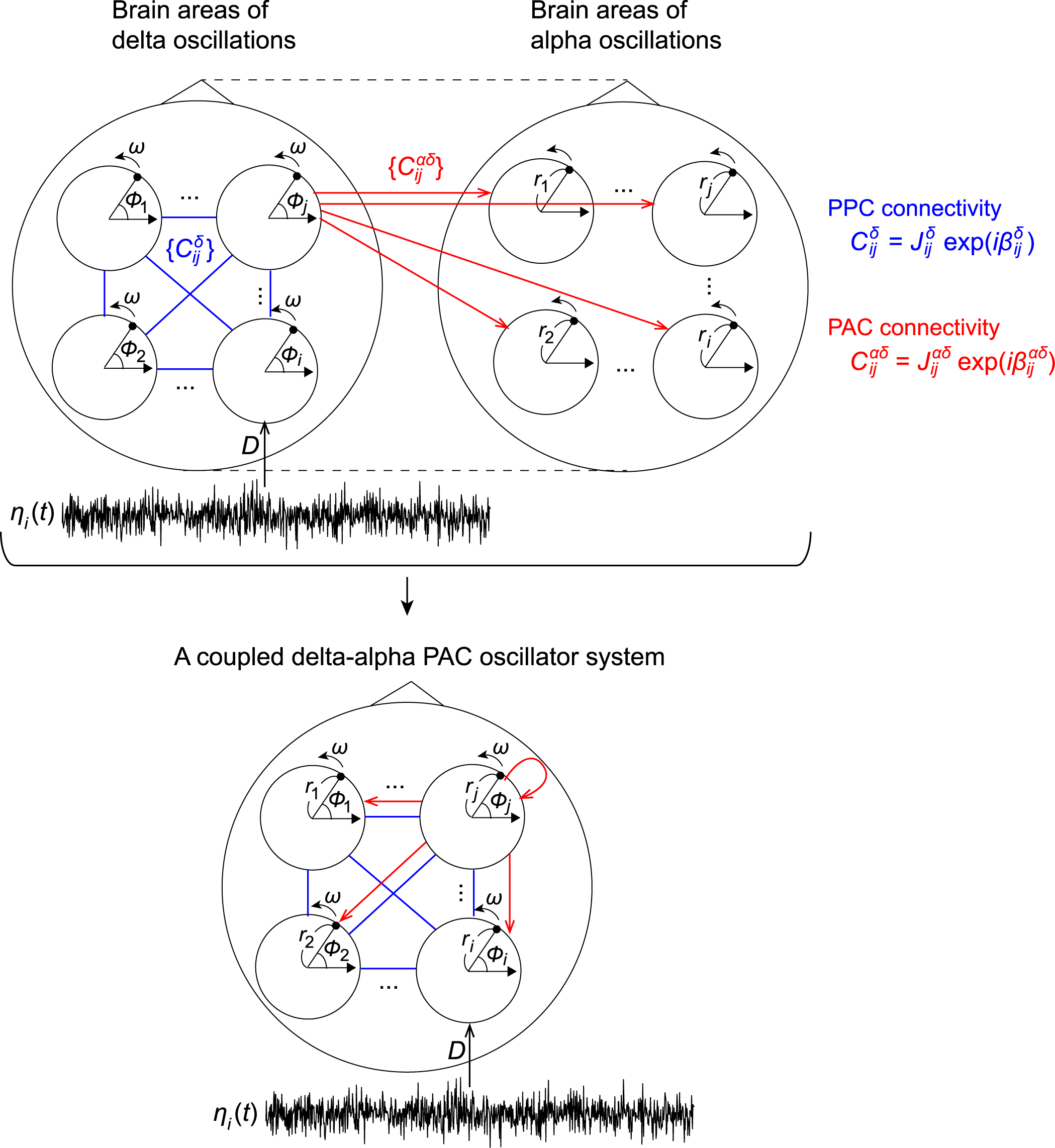

Supplement: S1 Fig — The model comprised N PAC oscillators whose phases {ϕi(t)} and amplitudes {ri(t)} corresponded to delta- and alpha-band activity, respectively. The phase ϕj(t) interacted with ϕi(t) and ri(t) via the PPC connectivity Cijδ=Jijδexp(iβijδ) and the PAC connectivity Cijαδ=Jijαδexp(iβijαδ). The phase ϕi(t) was driven by fluctuation ηi(t) with the level D for i = 1, 2, …, N. (TIF) [file pcbi.1008929.s001.tif]

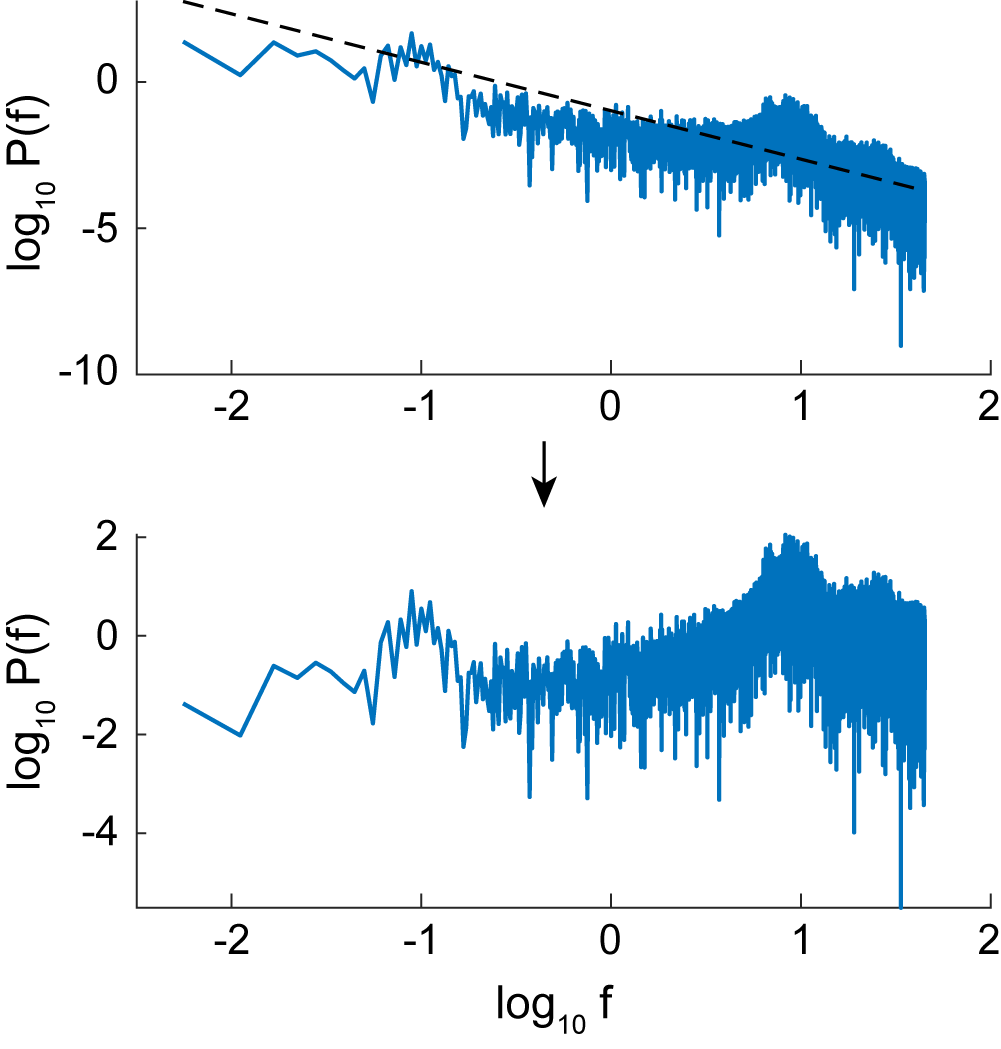

Supplement: S2 Fig — The power spectra of EEG signals were detrended in the double-logarithmic scale with respect to each signal. The detrended spectra were averaged, resulting in a single spectrum from which we estimated the peak frequency (refer to Fig 3E). (TIF) [file pcbi.1008929.s002.tif]

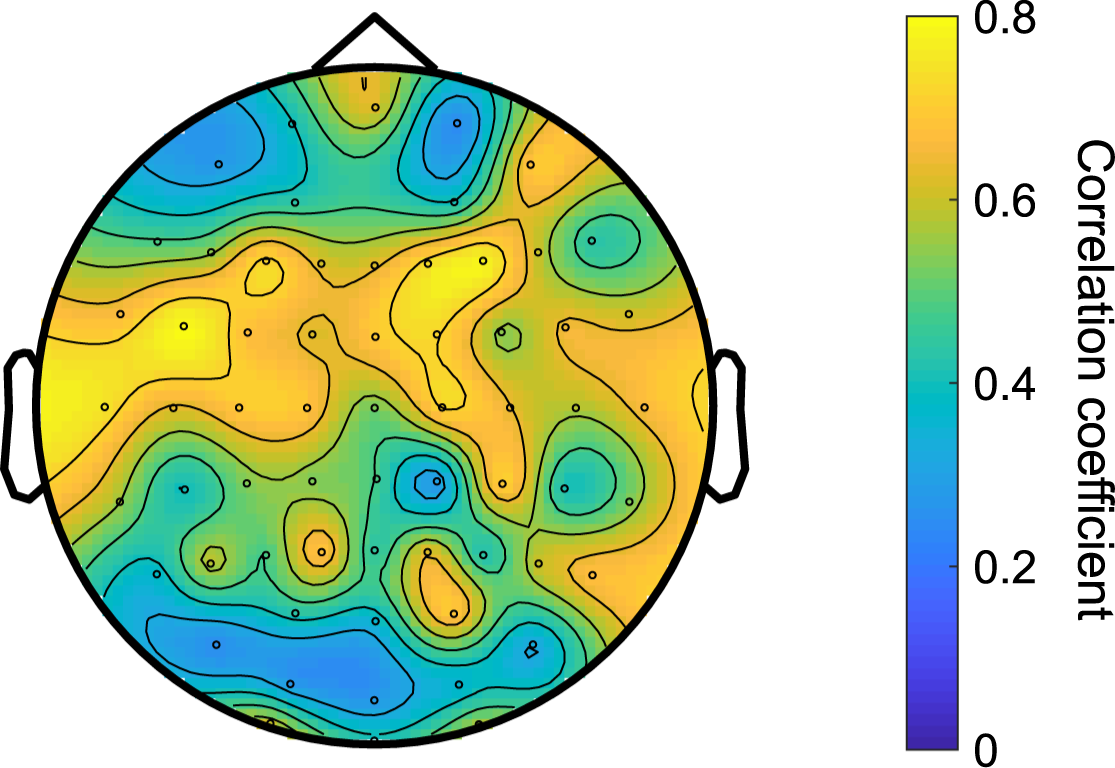

Supplement: S3 Fig — The MI of the EEG signals was estimated successively over time by a sliding time window with a length of the inverse of the delta-band peak frequency (i.e., one delta wave period). Significant correlations were shown from the scalp sites of more than 50 electrodes. (TIF) [file pcbi.1008929.s003.tif]

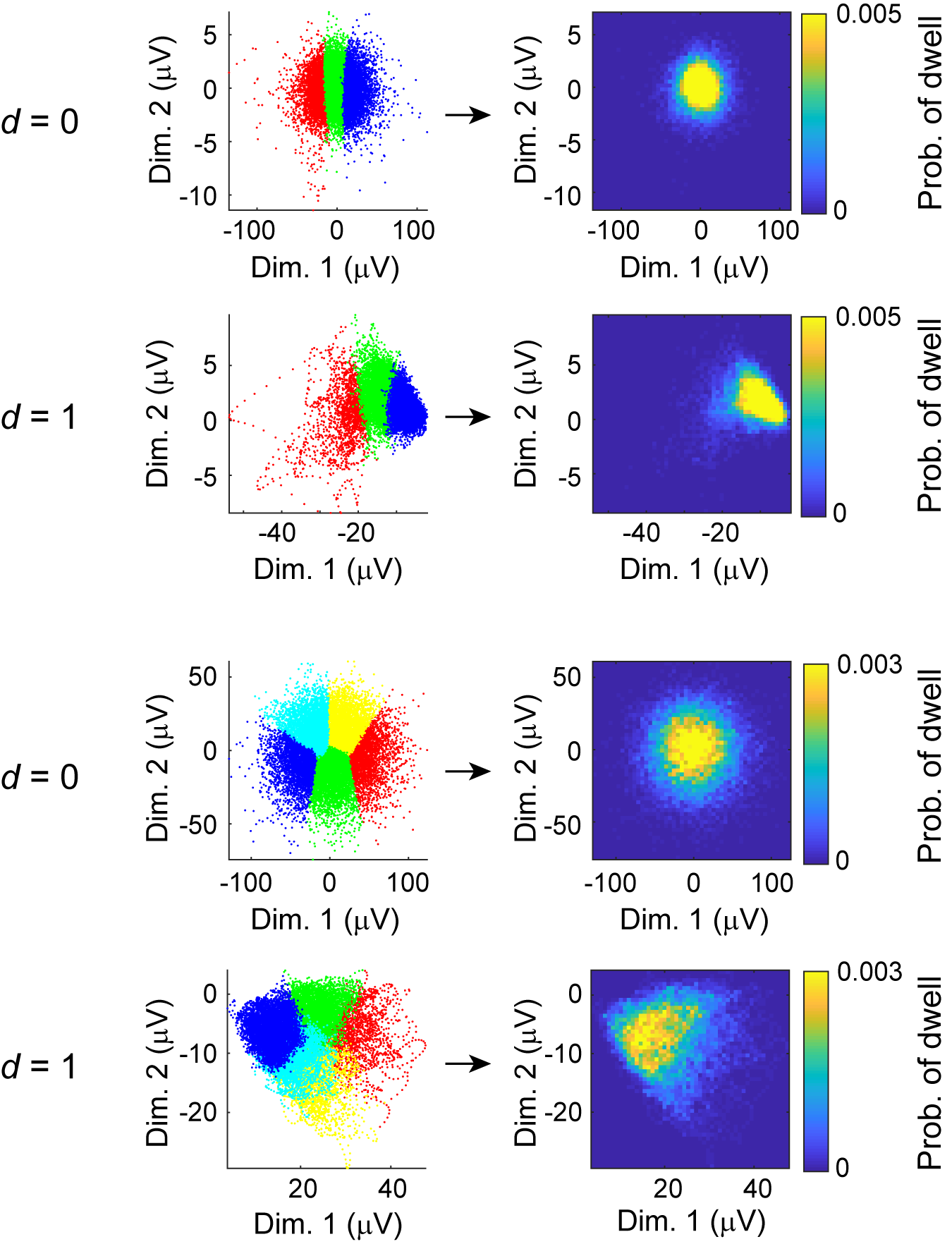

Supplement: S4 Fig — The surrogate data testing did not reject the null hypothesis H0 in condition d = 1 for all the experimental delta-alpha PAC dynamics identified in this study, and many of them were not rejected in the condition of d = 0. This Figure corresponds to Fig 4A to 4E, and depicts the case of K = 3 estimated from the condition d = 2 for comparison purposes. (TIF) [file pcbi.1008929.s004.tif]

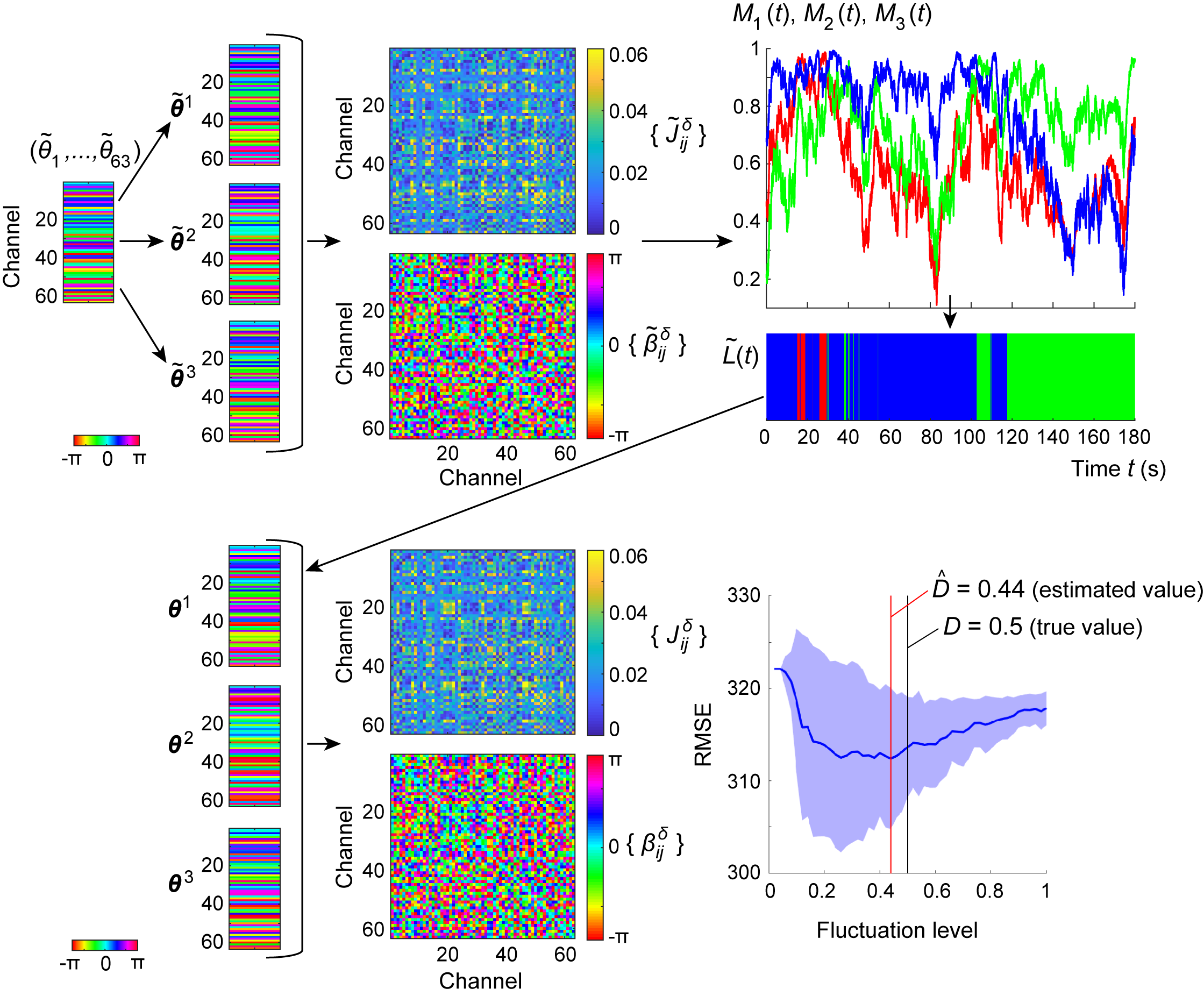

Supplement: S5 Fig — Artificially generated phase patterns {θ˜μ} and the estimated phase patterns {θμ} showed high similarities 0.967, 0.881, and 0.962, which were evaluated by (1/N)|∑j=1Nexp(i(θ˜jμ-θjμ))| for μ = 1, 2, 3, respectively. Accordingly, simulated PPC connectivity C˜δ and its estimation Cδ showed high cosine similarity (0.979 between the absolute parts and 0.636 between the argument parts). The estimated fluctuation level D^ was 0.44, which was close to the true value of D = 0.5. (TIF) [file pcbi.1008929.s005.tif]

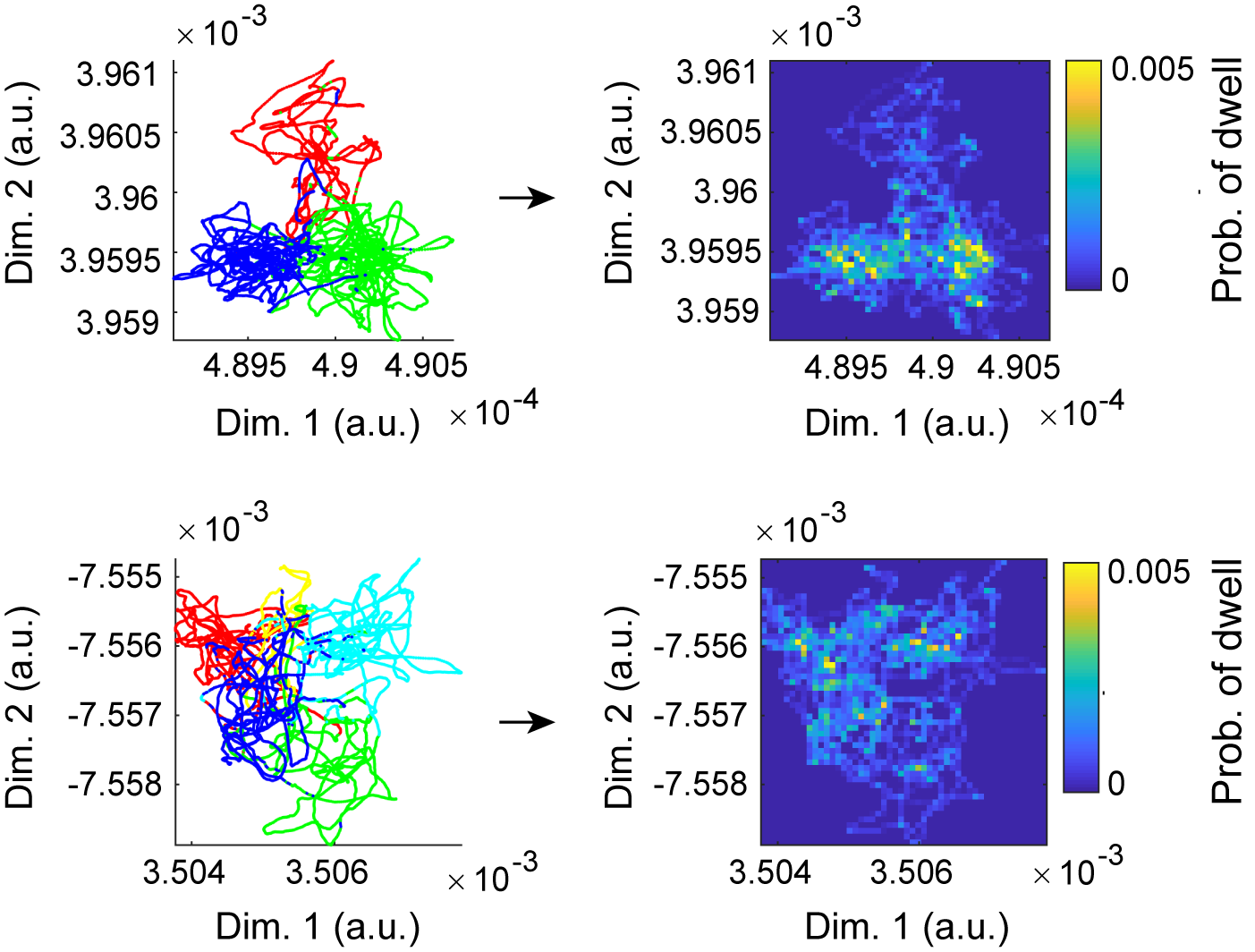

Supplement: S6 Fig — The surrogate data testing did not reject the null hypothesis H0 in condition d = 1 for all the modeled delta-alpha PAC dynamics. This Figure corresponds to Fig 8A to 8D, and depicts the case of K = 3 for comparison purposes. (TIF) [file pcbi.1008929.s006.tif]
